# Is dental plaque the only etiological factor in Amlodipine induced gingival overgrowth? A systematic review of evidence

**DOI:** 10.4317/jced.54715

**Published:** 2018-06-01

**Authors:** Sumit Gaur, Rupali Agnihotri

**Affiliations:** 1 MDS, Associate Professor, Department of Pedodontics and Preventive Dentistry, Manipal College of Dental Sciences, Manipal Academy of Higher Education, Manipal, Karnataka, India; 2 MDS, Associate Professor, Department of Periodontology, Manipal College of Dental Sciences, Manipal Academy of Higher Education, Manipal, Karnataka, India

## Abstract

**Background:**

Amlodipine, a dihydropyridine calcium channel blocker (CCB) is commonly prescribed for cardiovascular conditions. Its administration may produce an uncommon adverse oral manifestation, the gingival overgrowth (GO). Lately, there has been an increase in the rate of GO in patients on amlodipine therapy. The current systematic review was undertaken to evaluate the evidence on plausible risk factors involved in amlodipine induced gingival overgrowth (AIGO).

**Material and Methods:**

Literature search was conducted in the databases like Pubmed (Medline), Scopus and Google Scholar to include the original research articles related to etio-pathogenesis of AIGO.

**Results:**

About 270 documents were identified through primary search, of which 13 original research articles were included. Most common risk factor for AIGO was administration of amlodipine in subjects with poor plaque control. However, high dosage of drug, duration of therapy and inherent genetic susceptibility were recognized as other plausible risk factors.

**Conclusions:**

It was concluded that AIGO is no longer a rare phenomenon. It is therefore imperative for the physician to identify and inform patients, about the risk factors associated with the overgrowth at the initiation of therapy. This would prevent the development of GO’s and improve the patient’s quality of life.

** Key words:**Amlodipine, calcium channel blockers, gingival overgrowth, hypertension.

## Introduction

Hypertension, a “*silent killer*” is one of the most important modifiable risk factors for cardiovascular and associated conditions like stroke, dementia, ischemic heart disease, vision loss, heart and kidney failures (1). About one billion adults are hypertensive globally and this number may reach 1.56 billion by the year 2025, a sharp increase of about 60% from the year 2000 (2). The increasing prevalence of hypertension has been attributed to population overgrowth, ageing, behavioral and lifestyle associated risk factors. About 9.4 million people die every year because of hypertension and its complications and half of them are related to heart disease and stroke (3).

For decades, antihypertensive drugs including diuretics, alpha and beta blockers, angiotensin converting enzyme (ACE) inhibitors, angiotensin II type 1 receptor blockers (ARB) and calcium channel blockers (CCB’s) have been used to manage these conditions (1). They are administered either alone or in combination, depending on the needs of the patient.

The CCB’s are the most common antihypertensive agents, prescribed in about 37% of the cases. They comprise of two subclasses, dihydropyridines and non-dihydropyridines (2). Although their mechanism of action is the same, they have varied pharmacological effects. While the dihydropyridines are potent vasodilators, the non-dihydropyridines produce more negative intropic effects. The former were first introduced in 1960’s. Since then they have undergone several modifications to improve their efficacy and safety resulting in their four generations. The first-generation (e.g. nicardipine and nifedipine) were very effective but owing to their short duration and rapid vasodilator action, they produced more adverse effects. This was followed by the development of second (e.g. benidipine, and efonidipine), third (e.g. amlodipine and azelnidipine) and fourth (e.g. lercanidipine and lacidipine) generation agents which were relatively stable with less side effects.

Amongst the CCB’s, amlodipine, a third generation agent, is prescribed very frequently. It may be used either alone or in combination therapy. These medications often require lifelong administration, exposing the patients to side effects including adverse oral reactions like gingival overgrowths (GO).

**Figure 1:**
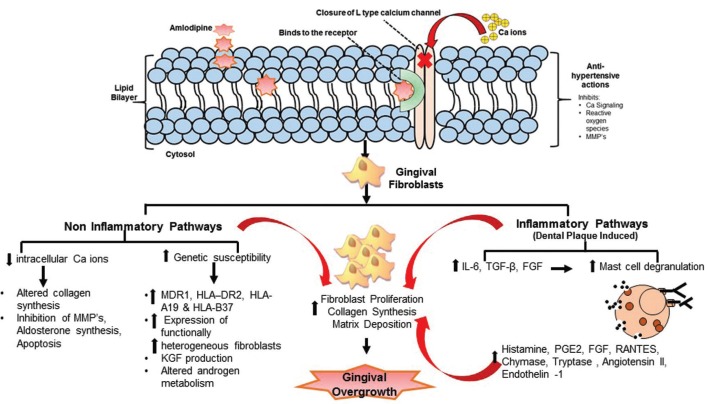
Mechanism of amlodipine induced gingival overgrowth.

In general, GO may be inflammatory (as a consequence of accumulation of bacterial dental plaque), idiopathic, related to systemic conditions like scurvy, leukemia, neoplasia, hormonal imbalances (e.g. puberty and pregnancy) or administration of medications. Currently, more than 20 prescription medications are associated with gingival enlargement. These include anticonvulsants (e.g. phenytoin), immunosuppressants (e.g. cyclosporine A) and CCB’s like nifedipine, diltiazem and verapamil (4). The prevalence of CCB induced GO was about 6-15% for nifedipine, 5-20% for diltiazem and 5% for verapamil (4). They were rarely seen with amlodipine and felodipine usage, until 1993, when the first case of amlodipine induced GO (AIGO) was reported (5). It has been suggested that age, genetics, drug variables and pre-existing gingival inflammation all influence the response of gingiva to these medications (6). They produce massive enlargements which may be localized or generalized. These subsequently interfere with routine oral hygiene practices and promote bacterial plaque accumulation and inflammation. Thus a vicious cycle is developed resulting in persistent overgrowths that complicate the masticatory function and esthetics. In the past, various mechanisms like disturbed fibroblastic proliferation and apoptosis as well as altered connective tissue hemostasis were implied in the pathogenesis of drug induced GO (DIGO) (Fig. 1).

Lately, there has been a rise in the number of reports related to GO in amlodipine treated cases (7). Although various mechanisms have been proposed, the exact role of amlodipine in GO is yet to be elucidated. With this background the current review aims to evaluate the evidence on plausible risk factors for this alleged rare occurrence.

## Material and Methods

The research question was formulated using the guidelines of Preferred Reporting Items for Systematic Reviews and Meta-Analysis (PRISMA) and according to the Participants, Interventions, Control, and Outcomes (PICO) principle.

a. Focused Question

The focused questions of interest was “Is dental plaque the only etiological factor in Amlodipine induced gingival overgrowth?”

b. Eligibility Criteria

Our search included longitudinal, case-control and cross-sectional studies to identify the various plausible risk factors for AIGO. Only free full text articles of these studies were included if they identified risk factors related to etiopathogensis of AIGO.

Exclusion criteria were animal studies, experimental studies, *in vitro* studies, reviews, case reports/series or studies involving pediatric patients, conference papers and documents published in a language other than English.

c. Literature Search strategy

Literature search was made to include the longitudinal, case-control and cross-sectional studies on AIGO. A systematic search of the electronic databases PubMed (Medline), Scopus and Google Scholar was performed from 2013 to January 2018 using a combination of keywords like Amlodipine, Gingiva, Gingival, Overgrowth, Enlargement and Hyperplasia. These terms were searched in title, abstracts or keywords. The titles and abstracts of retrieved studies were screened for eligibility by the authors and all irrelevant studies were excluded. The full texts of the articles were then read and assessed for inclusion.

d. Data extraction

The following data were extracted from the included articles by two independent authors using a standardized data collection form: authors and year of study, study design, number of subjects, mean age, drug variables (mean dosage, duration and frequency of amlodipine usage), periodontal variables (degree of GO, plaque index and gingival index) and the main outcomes.

Disagreements were resolved by discussion between the authors and a consensus was reached before including the studies in the review.

e. Assessment of quality

The critical appraisal of the included studies was performed using the criteria from Strengthening the Reporting of Observational studies in Epidemiology Statement (STROBE) (8). Following eight criteria were considered most important in the context of this review and were included in the checklist: reporting of study design, description of study participants, justification of sample size, inclusion of drug and periodontal variables, potential confounders, measurement of the outcomes and appropriate statistical analysis. Each criterion was given a response of either “Yes” or “NO”. Each study could have a maximum score of 8. After the scores were summed, the methodological quality was graded as low (0-3), acceptable (4-6), and high (7-8).

## Results

a. Study selection

**Figure 2:**
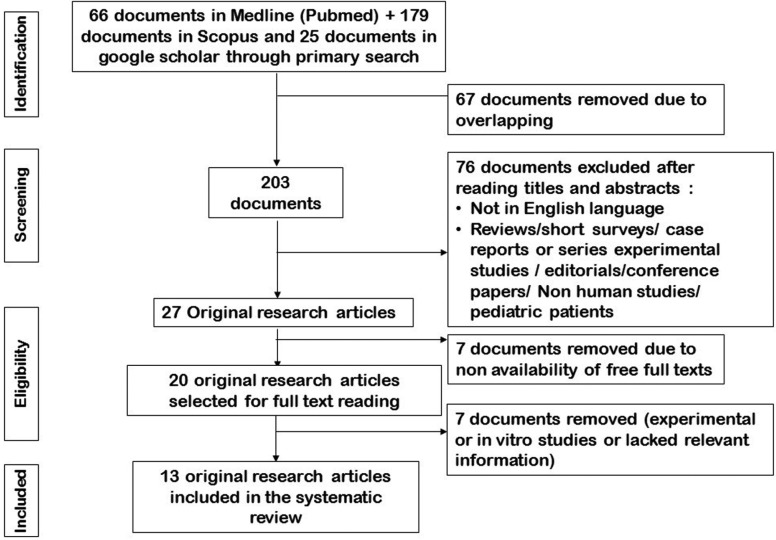
Search strategy for Original research articles.

The search strategy for identification of relevant studies following the PRISMA guidelines is presented in Figure 2. An initial search identified a total of 270 documents. Of these 67 documents were removed due to overlapping. Further screening of 203 documents resulted in 27 original research. Among them 7 documents were excluded due to non-availability of free full text. The full texts of 20 documents were studied in detail. Of these, 7 were excluded as they were either experimental or in vitro studies or lacked relevant information. Finally, 13 original research articles were included in the systematic review and processed for data extraction.

b. Characteristics of the original studies

The characteristics of the original research studies have been summarized in  Table 1,  Table 1 continue,  Table 1 continue-1. There were 10 cross- sectional (7,9-12,14,16,18-20) and 3 case control studies (13,15,17). About 7 studies were conducted in India (7,13-17,19) and one each in United States of America (9), United Kingdom (10), Japan (11), Germany (12), Sudan (18) and Nigeria (20). The total number of subjects involved in these studies ranged from 25 to 4290 with the number of males having AIGO being more than females. Some studies involved estimation of prevalence of amlodipine intake in patients on CCB’s and further evaluated the presence of GO (7,10,12,20). The mean age of the subjects on amlodipine therapy ranged between 30 to 87 years.

**Table 1:**
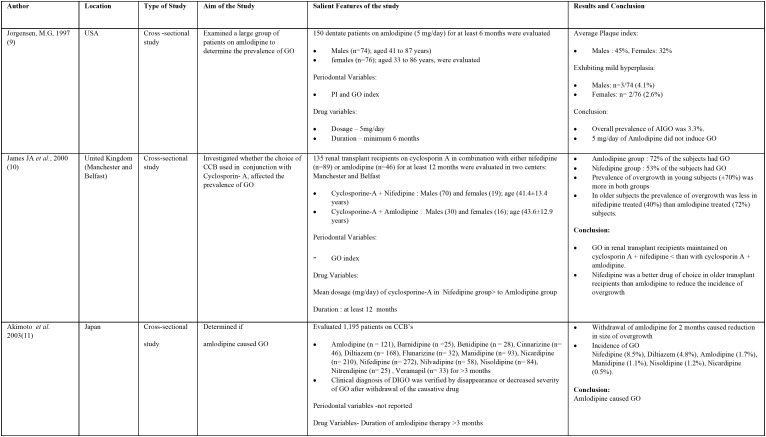
Characteristics of the original research studies included in systematic review.

**Table 1 continue:**
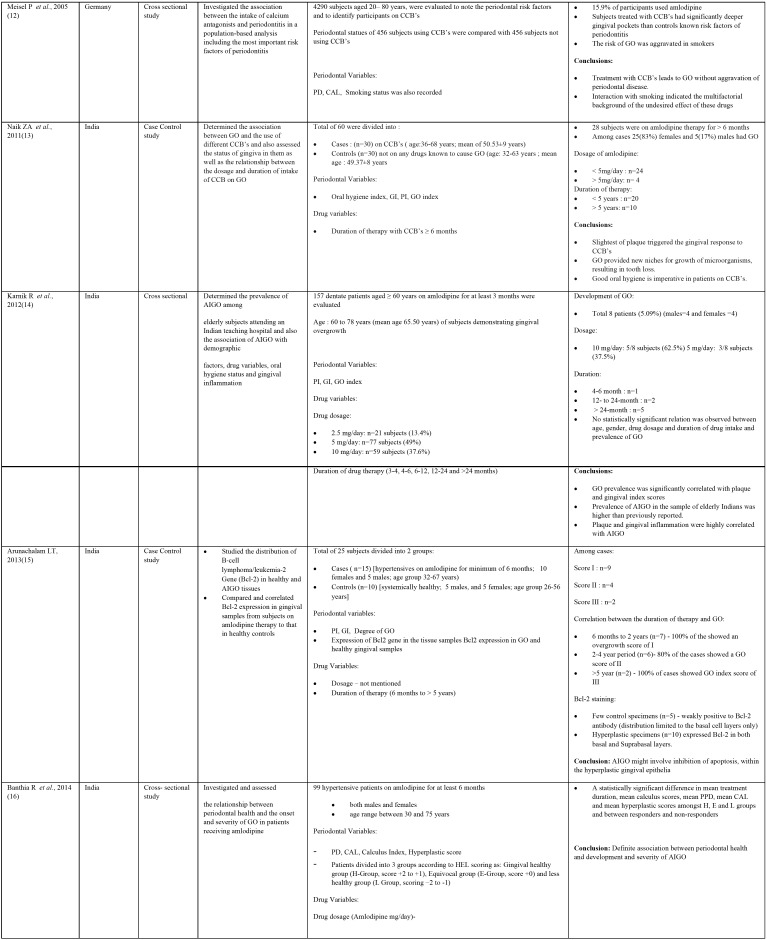
Characteristics of the original research studies included in systematic review.

**Table 1 continue-1:**
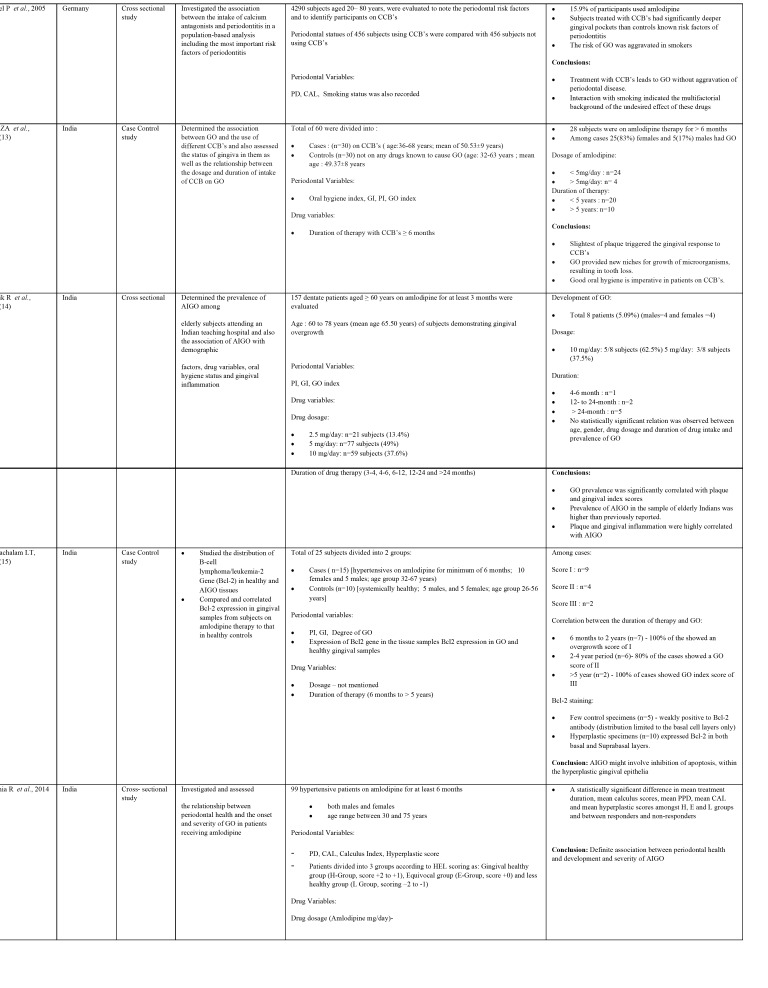
Characteristics of the original research studies included in systematic review.

c. Clinical Periodontal Parameters estimated

The plaque and other triggering factors for AIGO were measured with the help of Plaque index (7,9,13-15,17,20), Calculus index (12,16) and simplified oral hygiene index (13,18) in the included studies. The periodontal status was evaluated by measuring Gingival Index (7,13-15,17,20), Probing pocket depths (7,12,16) and Clinical attachment loss (12,16). Furthermore, different GO indices were utilized to measure the horizontal and vertical component of GO (7,9,10,13-20). Additionally, the expression of Bcl2 gene was observed in the histological samples of overgrown gingival tissues in one study (15). Other confounding factors like smoking were assessed in two studies (7,12).

d. Drug variables

The mean dosage of amlodipine causing GO ranged from 2.5 to 10 mg/day. Two studies reported 2.5mg/day dosage (14,19), five studies each reported 5mg/day (7,9,13,14,19) and 10mg/day (13,14,16,19,20) dosage therapy. One out of two studies reported GO with 2.5 mg/day dosage (19), while all five studies with 5mg/day (7,9,13,14,19) and 10 mg/day (13,14,16,19,20) therapy reported GO. The duration of amlodipine therapy ranged from 3 months to > 9 years. Four studies evaluated development of overgrowth with ≤ 6 months of therapy (9,11,14,15). However, majority of studies evaluated GO with > 6 months of therapy (7,10,13-16,19,20).

e. Main Outcomes

The risk factor for AIGO identified in majority of the studies was administration of amlodipine in patients with poor oral hygiene (7,13,14,16-18). A significant association of AIGO was seen between PI and GI in these studies. Two studies reported presence of GO in amlodipine patients but did not evaluate the levels of plaque and oral hygiene (11,20). The probing depth and CAL were associated with GO in two studies (12,16). Furthermore, smoking was suggested to promote GO independent of drug therapy in two studies (7,12). The dosage of the drug and duration were not significantly correlated with GO in three studies (7,14,19) while two studies reported probable association between the duration of therapy and AIGO (15,16). However, two studies, reported a reduction in size of GO following amlodipine withdrawal for two months (11,17). The lowest dose of drug causing GO was 2.5 mg/day in one study (19) while 10mg/day dosage resulted in rapid overgrowth (15). A minimum of 3 months of therapy resulted in overgrowth in one study (11). Interestingly, two studies reported increased incidence of GO in patients on amlodipine when compared to nifedipine (10,20). Furthermore, a study in geriatric renal transplant patients suggested that amlodipine had a greater potential to cause GO in elderly than nifedipine and the latter should be considered in elderly renal transplant patients (10). Two studies regarded smoking as an independent risk factor for AIGO (7,12).

The salient features of the studies and their outcomes have been summarized in  Table 1.

Arunachalam LT and Rao S in 2013 (15) studied the distribution of B‑cell lymphoma/leukemia‑2 Gene (Bcl‑2) in healthy and AIGO tissues and also compared and correlated its expression in gingival samples from subjects on amlodipine and healthy controls. Bcl-2 has been found to be associated with apoptosis which involves enhanced calcium influx or intracellular calcium re-localization and the calcium in the mitochondria. Bcl‑2 inhibits apoptosis by influencing the redistribution of intracellular calcium. It was found that few control specimens (n=5) were weakly positive to Bcl‑2 antibody (distribution limited to the basal cell layers only) while hyperplastic specimens (n=10) expressed Bcl‑2 in both basal and suprabasal layers. It was suggested that plausibly apoptosis of gingival keratinocytes was inhibited in low calcium conditions as seen with amlodipine administration, as sustained elevation of calcium was imperative to activate degradative enzymes such as calcium‑dependent protease and endonucleases responsible for DNA degradation. Besides, gingival keratinocytes grown under low levels of calcium expressed Bcl‑2, which inhibited apoptosis. In contrast, keratinocytes grown under high levels of calcium expressed Bax, which induced apoptosis. Additionally this study also associated duration of therapy with gingival overgrowth as it reported that seven cases showed mild overgrowth within 6 months to 2 years of therapy while six cases showed moderate GO within 2 to 4 years of therapy. Further, therapy for more than 5 years resulted in severe overgrowth in two cases. This could be related to prolonged exposure of tissues to amlodipine which enhanced the expression of Bcl2 and inhibited apoptosis of gingival keratinocytes.

Banthia R *et al.* in 2014 (16) investigated and assessed the relationship between periodontal health and onset and severity of GO in 99 patients on amlodipine. About 68 patients taking 8.46±2.33 mg/day of amlodipine developed GO in < 5 years of therapy. Additionally a strong association was seen between periodontal health and GO.

Gopal S *et al.*, 2015 conducted a cross-sectional study to determine the prevalence and risk factors for GO in subjects on CCB’s. Of the 133 subjects evaluated 102 were on amlodipine. Among them about 32 subjects developed GO. It was suggested that patients on anti-hypertensives had poor oral hygiene and gingival inflammation promoted development of DIGO.

f. Quality of the included studies

STROBE-based quality analysis showed a high (score of 7) for two studies (7,12) and low for one study (score 3) (11) while all the other studies had an acceptable scores of 5 (9,17) and 6 (10,13-16,18-20) ( Table 2). The most common shortcomings of all the studies were the lack of sample size calculation and failure to address the confounding factors. Furthermore, some did not report the dosage of amlodipine prescribed to all the patients. Duration of therapy and management were also inadequately reported. The overall quality of included studies for assessing risk factors for AIGO was fair. Nevertheless, small sample sizes and the failure to report the dosage and duration of amlodipine therapy limits the application of these study outcomes.

**Table 2:**
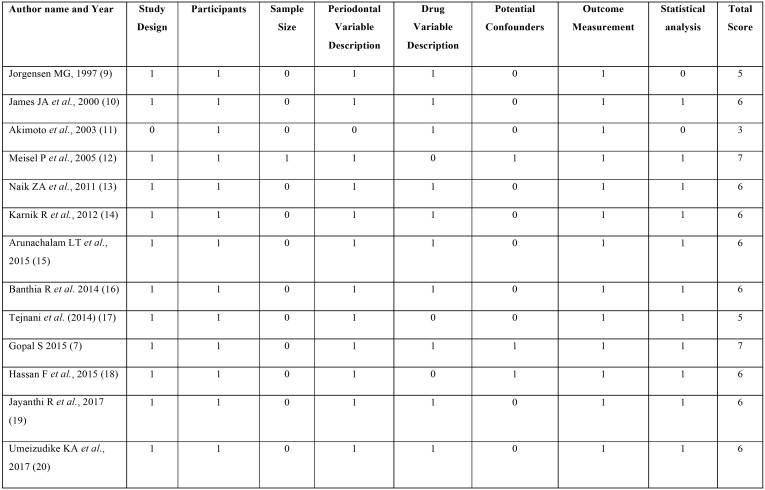
STROBE analysis for assessing quality of studies included in the systematic review.

## Discussion

For decades, amlodipine was regarded as a “safe drug” when compared to other CCB’s as it was associated with relatively few adverse reactions (21). This was mainly related to its longer duration of action requiring reduced dosage. Subsequently, there was an increase in its prescription rate for hypertension and angina pectoris which increased its side effects including GO. The present systematic review explored the association of various plausible risk factors for AIGO. Since most of this evidence has been reported in case control and cross sectional studies, this systematic review appraised their data to seek variables related to this purported rare occurrence.

In the past the prevalence of AIGO was 1.7% to 3.3% (21). Lately, the data suggests that there has been an increase in the number of these cases (7). As this CCB is the most preferred drug for long term maintenance of hypertensive and angina patients, it is worth understanding the various variables related to one of its major side effects, the GO. The pooled data in the present review revealed increased rate of GO (26.7%) among subjects on amlodipine.

The mean age of occurrence of GO in the present review was about fourth to fifth decade of life which was similar to previous studies (7). Although, there is no sexual predilection for DIGO, the prevalence of CCB induced GO has been shown to be higher in males than females (7). This was seen in the present review as well.

**Figure 3:**
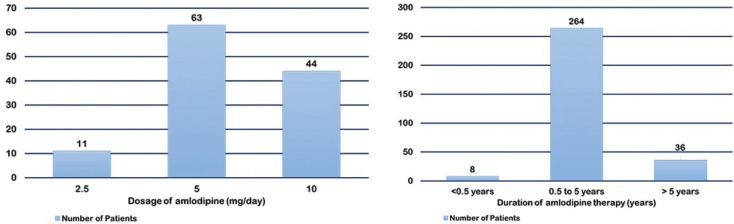
Total number of patients developing gingival overgrowth with various dosages and duration of the rapy with amlodipine.

Various drug variables like dosage, duration of therapy and concentration of drug in plasma and local fluids, like gingival crevicular fluid and saliva, play an important role in DIGO (6). According to Seymour, the dosage of drug is not a significant indicator of their effects on gingival tissue, rather it was more appropriate to relate it to patient’s body weight. Furthermore, their concentration in body fluids was also considered important in this aspect (6). It has been postulated that a certain threshold concentration of the drug or its metabolite was essential to “activate” gingival fibroblasts (22). However, increasing the levels of drug above this threshold did not affect the severity of the lesion. Besides, a direct correlation was observed between the concentration of drug in local fluids and DIGO. Although the mean dose of amlodipine reported to cause GO in the above studies was ranged from 2.5 to 10 mg, most of the subjects were on 5mg/day therapy (Fig. 3a). This was contrary to a previous study which suggested that 5mg/day of amlodipine could not result in GO (9). Besides, 10 mg/ day resulted in a more severe form of GO in all the cases in one study (15). Therefore, it may be suggested that dosage of drug may have an impact on GO and cannot be ignored, specifically in responders. The duration of therapy with amlodipine widely varied in this review with a minimum duration of six months in all the studies (Fig. 3b). One study reported a direct correlation between the degree of GO and duration of amlodipine therapy (15). Usually, the gingival manifestations of AIGO appear within the first three months of the drug administration (23) which was evident in one study in this review (11). Additionally, longer duration of therapy may increase the exposure of cells to amlodipine. This may enhance the expression of Bcl2, an antiapoptotic protein in cells in the presence of low calcium. Therefore, amlodipine could modify apoptosis of cells resulting in increased viability and growth (15). Subsequently, the cell cycle may be prolonged resulting in hyperplasia. The variation in the development of GO among the subjects could also be related to their inherent genetic susceptibility. Lately, multidrug resistant (MDR1) gene polymorphisms have been suggested to modify the inflammatory response to the drug (24). These subjects are classified as responders and special care should be taken while prescribing CCB’s in them. Besides, in two studies on renal transplant patients in the present review (10,18), amlodipine caused more GO. This may be related to altered pharmacokinetics of cyclosporine A by amlodipine (25).

Poor plaque control was identified as a risk factor in almost all the studies. This concurs with previous studies that proposed that GO hampered routine oral hygiene measures (6). Additionally, plaque accumulation caused inflammatory enlargement which superimposed the DIGO. It has been shown that CCB’s like nifedipine concentrate more in the areas of inflammation where they produce direct effects on gingival keratinocytes (26). Besides, amlodipine may inhibit apoptosis of human gingival fibroblasts in the presence of pro-inflammatory cytokines like TNF-α in inflamed gingival tissues (27). It may increase their viability and number leading to hyperplasia.

A combined non-surgical and surgical therapy with drug substitution is the most common treatment approach for DIGO. Although for AIGO non-surgical periodontal therapy without drug replacement may improve the condition (28), surgical excision is more reliable as it eliminates the hyperplastic tissue and promotes plaque control. Furthermore, it improves the esthetics. It is essential to substitute the drug to prevent any future recurrences. The most common drug used for substitution is an ACE inhibitor. Additionally, genetic susceptibility of the individual has to be recognized for successful treatment outcomes. Furthermore, it is imperative to educate the patients on maintenance of good oral hygiene through both personal and professional care. They should be informed about this uncommon side effect at the initiation of therapy to prevent complications.

The limitations of this review include moderate acceptability of the studies included. Furthermore, there were variations in the study designs, sample sizes, inadequate mea¬surement of drug and periodontal variables limiting the statistical analysis of the data. Longitudinal observational studies with exclusion of confounding variables are desirable to evaluate the role of factors other than plaque in AIGO.

## Conclusions

The results of this systematic review suggest that AIGO is no longer a rare occurrence. Plaque accumulation is an important risk factor for AIGO. Besides, inherent genetic susceptibility, drug dosage and duration of therapy are other important risk factors. As amlodipine is the most preferred drug for long term maintenance of hypertension and angina, it is imperative to explain this adverse effect to the patients. They should be informed about the meticulous plaque control required to prevent GO. Moreover, it is essential for the physician to recognize this condition and associated drug variables to effectively manage these cases. Combination therapy consisting of surgical and non-surgical periodontal therapy with drug substitution is the most reliable method for management of AIGO.
